# Filter forensics: microbiota recovery from residential HVAC filters

**DOI:** 10.1186/s40168-018-0407-6

**Published:** 2018-01-30

**Authors:** Juan P. Maestre, Wiley Jennings, Dennis Wylie, Sharon D. Horner, Jeffrey Siegel, Kerry A. Kinney

**Affiliations:** 1 Department of Civil, Architectural and Environmental Engineering, The University of Texas at Austin, Austin, TX USA; 2 School of Nursing, The University of Texas at Austin, Austin, TX USA; 3 Department of Civil Engineering, University of Toronto, Ontario, Canada; 4 Dalla Lana School of Public Health, University of Toronto, Ontario, Canada; 5 Genome Sequencing and Analysis Facility, The University of Texas at Austin Center for Biomedical Research Support, The University of Texas at Austin, Austin, TX USA

**Keywords:** Built environment, Indoor microbiology, Filter forensics, Bacteria, Fungi, HVAC

## Abstract

**Background:**

Establishing reliable methods for assessing the microbiome within the built environment is critical for understanding the impact of biological exposures on human health. High-throughput DNA sequencing of dust samples provides valuable insights into the microbiome present in human-occupied spaces. However, the effect that different sampling methods have on the microbial community recovered from dust samples is not well understood across sample types. Heating, ventilation, and air conditioning (HVAC) filters hold promise as long-term, spatially integrated, high volume samplers to characterize the airborne microbiome in homes and other climate-controlled spaces. In this study, the effect that dust recovery method (i.e., cut and elution, swabbing, or vacuuming) has on the microbial community structure, membership, and repeatability inferred by Illumina sequencing was evaluated.

**Results:**

The results indicate that vacuum samples captured higher quantities of total, bacterial, and fungal DNA than swab or cut samples. Repeated swab and vacuum samples collected from the same filter were less variable than cut samples with respect to both quantitative DNA recovery and bacterial community structure. Vacuum samples captured substantially greater bacterial diversity than the other methods, whereas fungal diversity was similar across all three methods. Vacuum and swab samples of HVAC filter dust were repeatable and generally superior to cut samples. Nevertheless, the contribution of environmental and human sources to the bacterial and fungal communities recovered via each sampling method was generally consistent across the methods investigated.

**Conclusions:**

Dust recovery methodologies have been shown to affect the recovery, repeatability, structure, and membership of microbial communities recovered from dust samples in the built environment. The results of this study are directly applicable to indoor microbiota studies utilizing the filter forensics approach. More broadly, this study provides a better understanding of the microbial community variability attributable to sampling methodology and helps inform interpretation of data collected from other types of dust samples collected from indoor environments.

**Electronic supplementary material:**

The online version of this article (10.1186/s40168-018-0407-6) contains supplementary material, which is available to authorized users.

## Background

People in the USA spend an estimated 80–90% of their time indoors [1–3], much of it at home. The potential health impacts of chemical and biological exposures that occur in the home have been studied for decades [4–13]. Recent advances in high-throughput DNA sequencing technology [14–18] have spurred investigations into the relationship between the human microbiome and the microbiome present in homes, offices, and schools [10, 19–33]. Several recent molecular-based studies have also linked indoor microbial community exposures to human health outcomes, such as the development [34, 35] and severity [36] of childhood asthma as well as immune response to allergens [37].

The most common method used to delineate microbial communities in a home is to collect settled dust samples from dust reservoirs, such as door trims [21, 23], chairs [36], floors [34], and mattresses [35]. However, settled dust collects over an unknown period of time and these dust samples may be biased towards larger particles that settle quickly in the indoor environment [38]. Alternatives to sampling settled dust on home surfaces include using petri dishes and other passive materials to capture airborne particles as they settle [19, 30, 39], or short-term air samples collected on filters or in liquid impingers. These alternative techniques are advantageous because collection time can be determined, and the particles collected were necessarily airborne at the time of sampling. However, depending on the method employed, these methods may recover low biomass quantities that can limit their suitability for DNA-intensive analyses, such as simultaneous sequencing of bacterial, fungal, and viral communities from a single sample, metagenomic sequencing, and/or multiple polymerase chain reaction (PCR) and quantitative PCR (qPCR) assays. Additionally, they represent a temporal and spatial snapshot of airborne concentrations that may not be representative of other spaces in the building or a different sampling period [40].

Heating, ventilation, and air conditioning (HVAC) filters have also been successfully used to characterize the microbiota of indoor environments [41, 42]. This filter forensics approach uses the HVAC filters installed in homes as integrated, long-term samplers of particle-bound contaminants, such as microorganisms, allergens, SVOCS, and metals [30, 43–46]. The potential advantages of using HVAC filter dust are as follows: (a) most central HVAC systems have a filter, (b) the filters are in place for long, potentially known periods of time, and (c) they can collect particles from a wide spatial area, acting in essence as a high volume air sampler [45]. When combined with HVAC system characterization including system run-times and volumetric flow rates, HVAC filter dust sampling offers a spatially and temporally integrated way of detecting and assessing indoor air contaminants present at low concentrations in homes, a process referred to as quantitative filter forensics (QFF) [40].

One of the key challenges in QFF is understanding the reliability and repeatability of this approach as a method for examining the indoor microbiome. There are several interconnected factors that affect the results of the method including the representativeness of the microbial concentration of interest in the air that flows through the filter, the spatial distribution heterogeneity of the microbes across the face of the filter, the extraction of the dust containing the microbes from the filter, and the processing and analysis of the collected dust [40]. This paper focuses on the sampling and extraction of dust from the filter as this is a central challenge to HVAC sampling and the approach can introduce both bias and uncertainty in QFF. Common approaches to extraction include liquid-based extraction involving sonication, vortexing, and filtering (e.g., [38, 47]), swabbing (e.g., [48]), and vacuuming (e.g., [49]). These techniques may vary in several regards, including repeatability and biomass recovered. Since microbial communities [50, 51] and other health-relevant biological material [52] in indoor environments may vary significantly depending on the type of sampler employed, understanding these differences is important. Checinska and colleagues [53] employed two techniques to examine the microbiota of the international space station: analyzing dust from vacuum bags that had been used to sample from the protective screen on HEPA filters and removing particles directly from the HEPA filter with a scalpel. They found considerable differences in the taxonomy and viability of these two samples, likely because of differences in particle size collected by the two techniques, suggesting that sampling and extraction methods can strongly impact the microbiota that is recovered with this technique.

In this investigation, we perform repeated, randomized sampling without replacement of the dust on a single HVAC filter in order to compare three extraction methods (swabbing, vacuuming, and liquid-based). Both the dust matrix (the HVAC filter) and the post-extraction analysis of the microbial communities and quantities were standardized to allow for an explicit comparison of the different extraction techniques. The central questions being addressed are the within-approach variation which combines both the inherent variability in each of the extraction techniques and any spatial heterogeneity in the microbiota across the surface of the filter and the between-approach variation which looks at the differences in microbiota that result from different extraction approaches. This information is essential to the practical use of HVAC filter sampling for assessing the indoor microbiota as well as the ability to compare microbiotas from HVAC filter samples to other dust sampling approaches.

## Methods

### Filter installation and collection

This study is part of the Healthy Homes investigation (HUD: TXHHU0023-13), in which 60 households were recruited based on a resident child’s asthma status. All participants gave approval to participate in this study, in accordance with the University of Texas at Austin IRB Protocol # 2013-11-0026. Briefly, all homes evaluated were located in rural areas of central Texas and were sampled in both summer and winter to examine potential relationships between asthma severity and indoor microbial and chemical exposures as measured on home HVAC filters and in settled dust. Brand-new HVAC filters (multiple manufacturers, all ASHRAE Standard 52.2-2007 MERV 7-8) were installed for 30–45 days. These filters have an efficiency of 50–85% for 3–10 μm particles when tested according to ASHRAE Standard 52.2 and are generally used to prevent fouling of HVAC equipment for larger particles. The study presented in this paper comprises an in-depth investigation of five HVAC filters installed for 32 ± 2 days in a subsample of the homes in the larger investigation. The filters were located in return grilles on the ceiling or high on the wall in the subject homes. After removal, filters were sealed in anti-static bags and transported back to Austin, TX and stored at 4 °C until laboratory processing. Low-temperature conditions limit the ability of the microorganisms to reproduce on the filter after removal from the HVAC system [54, 55]. Sterile techniques were used for handling the filters in the field and lab.

### HVAC filter dust sampling

In this study, three techniques for removing dust from HVAC filters were investigated: (1) swabbing the surface of the filter, (2) vacuuming the surface of the filter, and (3) cutting pieces of the filter, extracting the dust in a buffer solution, and then filtering. For each of the three techniques, seven samples were collected from the HVAC filter being investigated. Each sample consisted of a composite of five 2.5 × 2.5 cm squares randomly selected from across the filter. To generate a random selection, a 2.5 × 2.5 cm grid was superimposed on the HVAC filters, and each square was assigned coordinates. A random number generator without replacement was then used to determine the selection of each subsequent square. In this way, a total of 35 squares were sampled per technique. No part of the filter was sampled more than once, and squares adjacent to vacuumed squares were not used for cut samples.

For each swab sample, a single phosphate-buffered saline Tween-20 (PBST) wetted swab (Floq Swab, Copan, Murrieta, CA) was used. Specifically, each of the five randomly selected gridded squares on the filter surface was swabbed for 5 s with the same swab. Swabs were then transferred directly to bead beating tubes (Mobio, East Palo Alto, CA) for DNA extraction. For each vacuum sample, a vacuum thimble was inserted into a clean thermoset plastic nozzle (Indoor Biotechnologies, Charlottesville, VA) attached to a vacuum cleaner (Genie Voltaire, Manchester NH). The collected dust cake was then transferred from the thimble to the same bead beating tubes. For each cut sample, five squares were cut from the filter and transferred to a pre-sterilized phosphate buffer solution (10 g/L NaCl, 0.25 g/L KCl, 1.43 g/L Na_2_HPO_4_, 0.25 g/L KH_2_PO_4_, DNA-free water, pH 7.0) in a sterile 50-mL centrifuge tube (Thermo Fisher Scientific Inc., Waltham, MA). The solution was sonicated and vortexed for 10 min, and then pre-filtered through a 20-μm pore size cellulose filter (Whatman Ltd., Maidstone, UK). The filtered solution was then vacuum-filtered through a 0.2-μm hydrophobic filter (Millipore, Billerica MA). Finally, the filter was transferred to the aforementioned bead beating tubes.

Sampling negative controls were included in the study to account for background materials and reagent contamination. These negative controls were obtained by processing an unused swab, an unused thimble (thimble plastic was cut out and placed in the bead beating tube), and an unused new HVAC filter for swab, vacuum, and cut samples, respectively.

### DNA extraction, PCR, and sequencing

DNA extraction was conducted as described previously [38]. Briefly, the swabs, the filter cake from the thimble (in the case of the vacuumed samples), and the 0.2-μm filter (in the case of the cuts) were added along with 100 μL lysozyme (3 mg/mL) and 300 μL phenol-chloroform-isoamyl alcohol (25:24:1) to a bead beating tube (lysing beads with 750 μL lysing solution) provided in the PowerSoil DNA Isolation Kit (Mo-Bio Laboratories Inc., Carlsbad, CA). Cell lysis by multidirectional beating was conducted in the FastPrep-24 homogenizer (MP Biomedicals LLC, Solon, OH), following manufacturer recommendations of 30 s at 5.0 m/s. DNA was eluted in 50 μL solution C6, quantified using Quant-iT PicoGreen dsDNA assay kit (Invitrogen Life Technologies, Grand Island, NY), diluted to equimolar aliquots, and stored at − 20 °C until sequencing.

Bacterial and fungal DNA were analyzed at the Genomic Sequencing and Analysis Facility (GSAF) at the University of Texas at Austin (Austin, TX, USA) for Illumina® paired-end (2 × 250) sequencing on the MiSeq platform. For bacteria, first-round PCR (19 cycles) was used to amplify the V4/V5 regions of the 16S rRNA gene using the primers 515F (5′-GTGYCAGCMGCCGCGGTA-3′) [56] and 909R (5′-CCCCGYCAATTCMTTTRAGT-3′) [57]. For fungi, first-round PCR (12 cycles) was used to amplify the ITS-1 region of the fungal nuclear-encoded ribosomal RNA genes (rDNA) using the primers ITS1F (5′-CTTGGTCATTTAGAGGAAGTAA-3′) [58] and ITS2 (5′-GCTGCGTTCTTCATCGATGC-3′) [59]. Primers included appropriate Illumina adapters with reverse primers also having an error correcting 12-bp barcode unique to each sample to permit multiplexing of samples. After PCR amplification, samples were prepared for their Illumina® sequencing run. This first round of PCR amplification was run in triplicate for each sample, pooled, and then cleaned using AMPure beads (New England Biolabs, Ipswich, MA). A second round PCR amplification (11 cycles for bacteria and 7 cycles for fungi) was performed with hybrid primers that added sample-specific barcodes. Both rounds of PCR amplification used Taq polymerase NEB Q5 (New England Biolabs, Ipswich, MA). The final PCR products for each sample after both rounds of amplification were again size-purified by removing amplicons less than 300 bp in length using AMPure beads (New England Biolabs, Ipswich, MA) and quantified using PicoGreen (Life Technologies, Carlsbad, CA). Samples were then normalized by amplicon mass and pooled for the Illumina® run. In addition, a random subset of samples was assessed on an Agilent BioAnalyzer (Agilent Technologies, Santa Clara, CA) to ensure correct amplicon size. Negative PCR controls (negative template) were included to test for contamination during amplification and sequencing processes. However, no sequences were obtained from these controls.

### Sequence processing and statistical analysis

Bacterial and fungal DNA sequences were processed and analyzed in QIIME v.1.8 [60] and FHiTINGS, version 1.3 [61]. Sequences were demultiplexed and forward and reverse reads were merged using FLASH v.1.2.11 [62] with maximum overlap of 250 bp. Sequences were quality-filtered (-q 19), and chimeras were removed via QIIME and USEARCH [63]. High-quality sequences were clustered into operational taxonomic units (OTUs) at 97% similarity using QIIME’s USEARCH-based open-reference OTU clustering workflow (pick_open_reference_otus.py). Global singleton OTUs were removed, and OTU proportions were standardized to the total number of high-quality reads. Taxonomy was assigned using the Ribosomal Database Project classifier [64] with the reference database Greengenes13_8 16s rRNA [65] for bacteria, and UNITE [66] for fungi (ITS_v7 _2015_08_01). In order to mitigate the influence of background DNA from the HVAC filter, reagents, or sample processing, reads obtained for each of the OTUs observed in the negative controls were subtracted from the corresponding OTUs in each respective sample type. All samples were rarefied to the least number of sequences present in any individual sample as is commonly done in microbiome studies.

All statistical analyses were performed in the R environment (www.r-project.org). Pair-wise dissimilarities between communities were calculated using weighted UniFrac [67]. Microbial community analysis of variance (implemented as ADONIS) and dispersion (betadisper) as well as mantel tests employed the Vegan package in R [68].

### Microbial DNA quantification

All qPCR reactions (samples, controls, and standards) were run in triplicate on an Applied Biosystems ViiaTM 7 Real-Time PCR System (Applied Biosystems, Foster City, CA) in 96-well plates. All samples were diluted 1:100 to avoid PCR inhibition. Bacterial 16S rRNA gene qPCR was conducted with a method modified from [69] which amplifies a conserved region of the 16S rRNA gene with a length of approximately 340 bp. The primers used for this assay were 1055F (5′-ATGGCTGTCGTCAGCT-3′) and 1392R (5′-ACGGGCGGTGTGTAC-3′), and the probe 16STaq1115 (5′HEX-CAACGAGCGCAACCC-TAMRA-3′). Each 20-μL reaction consisted of 10 μL 2X Taqman® Universal Master Mix (Applied Biosystems, Foster City, CA), 0.08 μL 50× BSA, primers and probe to final concentrations of 0.25 μM, and 2 μL template DNA.

Fungal 18S rRNA gene qPCR was conducted with a method modified from [70], which amplifies a region of the 18S rRNA gene that is conserved with a length of approximately 425 bp. The primers used for this assay were FF2 (5′-GCATCGATGAAGAACGCAG-3′) and FR1 (5′-TCCTCCGCTTATTGATATGC-3′). Each 10-μL reaction consisted of 5 μL 2X SYBR® Select Master Mix (Applied Biosystems, Foster City, CA), 0.5 ng/μL each primer, 1.6 μL 1× BSA, and 2 μL template DNA. The qPCR assay included an enzyme activation step of 50 °C for 2 min and an initial denaturation at 94 °C for 10 min, followed by, in the case of bacteria, 40 cycles of 94 °C for 30 s, 50 °C for 60 s, and 72 °C for 45 s; and in the case of fungi, 40 cycles of 94 °C for 60 s, 52 °C for 60 s, and 72 °C for 120 s with a subsequent fluorescence plate read. A melting curve was constructed at the end of each fungal qPCR run to ensure specificity in each sample’s amplification.

Every qPCR reaction included a standard curve. Standard curves were generated using 10-fold dilutions of genomic DNA from *Escherichia coli O157* and *Aspergillus niger* and were used to estimate the genome copy number in each qPCR reaction. For the conversion to bacterial and fungal genome quantities, the standard calibration curves accounted for the 54 rRNA operon copies per genome in *A. niger* and seven rRNA operon copies per genome in *E. coli O157*. Genome copy numbers are given on a per unit filter area basis for all samples and also on a per unit mass of dust basis for the vacuum samples. Measuring the dust mass recovered by swab and cut methods, which are both wet methods, was not performed in this study.

## Results

### Quantitative DNA recovery

All three techniques were evaluated for their ability to recover total, bacterial, and fungal DNA from the HVAC filter. Table 1 displays the DNA measured by qPCR in terms of both mass and genome copy number (GCN) per HVAC filter area. Note that the actual bacterial and fungal quantities present in the samples might have been underestimated given that not all microbial species will hybridize to any given set of universal primers and/or probes. In all cases, vacuum samples yielded a higher density of total, bacterial, and fungal DNA, followed by swabs and cuts. All three techniques also differed in their repeatability, as measured by the coefficient of variation (standard deviation/mean) for seven replicate samples. By this measure, swab samples were the most repeatable (CVbac = 0.31 with 95% confidence interval (CI) of (0.16, 0.44) estimated by bootstrapping (10,000 simulations); CVfun = 0.32 with 95% CI (0.14, 0.43)), followed by vacuum (CVbac = 0.35 with 95% CI (0.15, 0.47); CVfun = 0.42 with 95% CI of (0.20, 0.53)), and finally cut samples (CVbac = 1.24 with 95% CI (0.43, 1.58); CVfun = 1.86 with 95% CI (0.38, 2.07)). Bootstrapped 95% CIs for the differences in CV between cut samples and swab samples excluded 0 for both bacteria and fungi, while for the differences between cut samples and vacuum samples a bootstrapped 95% CI excluded 0 for bacteria but only a 90% CI excluded 0 for fungi. The difference in estimated CVs between swab and vacuum samples were not significant for bacteria or fungi. For all three techniques, the area density of bacterial genomes was higher than that of fungal genomes. Furthermore, vacuum, swab, and cut samples varied in their bacteria to fungi mass ratios (ANOVA, *p* < 0.001), with cut samples producing the largest ratios (Additional file 1: Figure S1). The mean bacterial and fungal genome copy numbers per mg of dust (non-sieved) obtained from vacuum samples were 10,084 ± 3522 and 57 ± 26, respectively. Since swab and cut samples are not amenable to mass analysis (due to the difficulty of measuring mass recovery), values per unit mass are not displayed.

**Table 1:** DNA and genome copy numbers measured by the three sampling techniques

Sampling technique	Median total DNA^a^ (min-max) (ng/cm ^2^)	Median bacterial DNA^b^ (min-max) (ng/cm^2^)	Median fungal DNA^b^ (min-max) (ng/cm^2^)	Median bacterial genome copies^b^ (min-max) (GCN/cm^2^)	Median Fungal Genome copies^b^ (min-max) (GCN/cm^2^)
Cut	0.718 (0.638–1.764)	0.02 (0.005–0.119)	0.002 (0.001–0.043)	324 (82–1773)	1 (0.34–19)
Vacuum	4.804 (2.980–12.787)	0.597 (0.311–0.953)	0.180 (0.103–0.341)	15,380 (7901–24,797)	79 (45–150)
Swab	1.523 (0.978–1.898)	0.040 (0.022–0.060)	0.020 (0.010–0.027)	623 (344–916)	9 (4–12)

Note that *n* = 7 for each of the sampling techniques

^a^Measured using Quant-iT™ PicoGreen® dsDNA Assay Kit

^b^Measured using qPCR methods

### Bacterial and fungal within-sample diversity

**Fig. 1:**
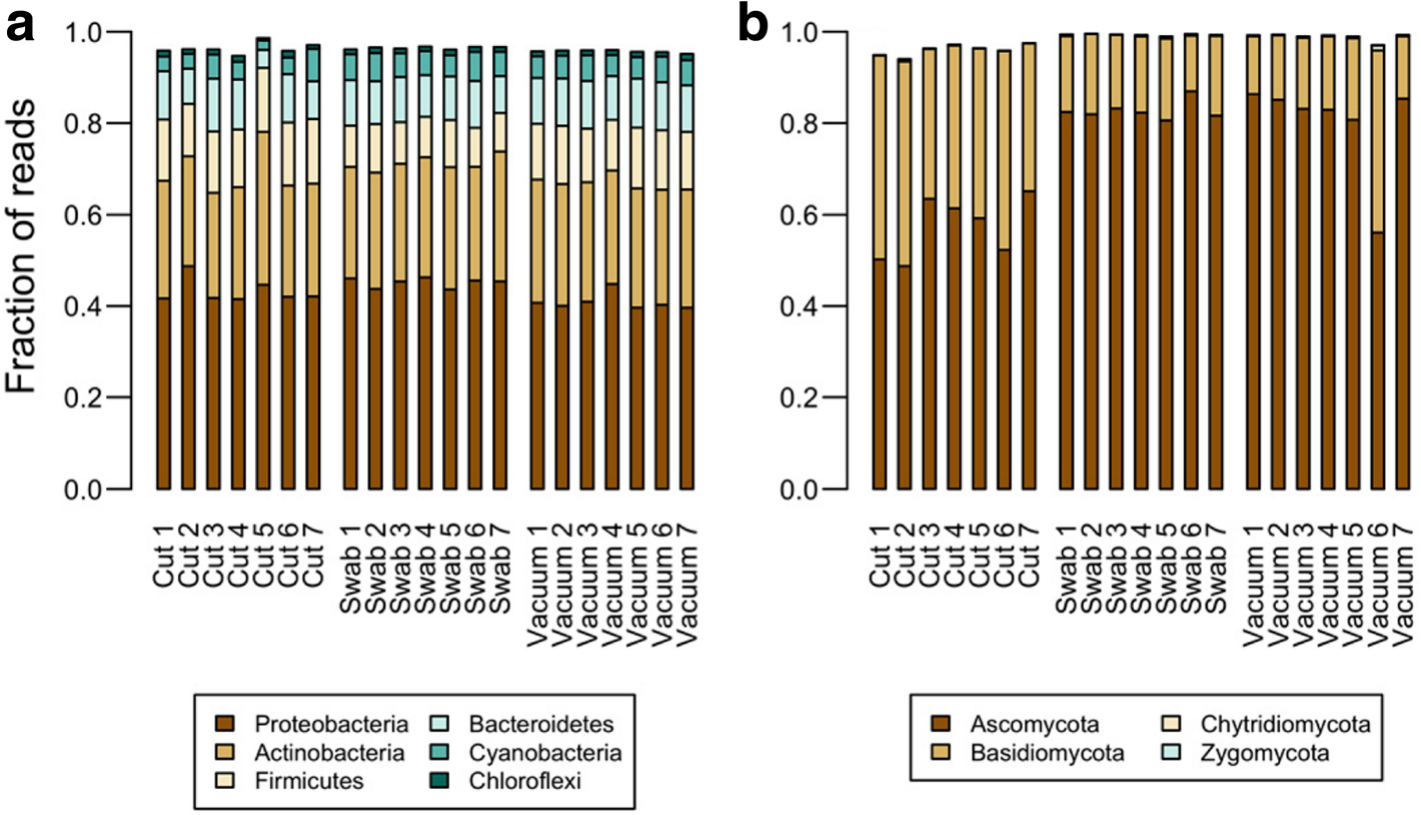
Relative abundance of top bacterial phyla (**a**) and fungal phyla (**b**) per sample. Selected taxa were top six groups with fully identifiable phylogeny and were ranked by median abundance

After quality filtering, a total of 2,913,611 high-quality bacterial sequences were clustered into 40,563 OTUs. Median sequences generated per sample were 81,986 for vacuum samples, 92,134 for swab samples, and 146,620 for cut samples. All samples were rarefied to 51,290 reads, yielding a total of 36,265 distinct OTUs. In total, two archaeal and 39 bacterial phyla were detected. Bacteria contributed an average of 99.9% of the sequences obtained with the V4–V5 region primer set, while Archaea contributed 0.06%, and < 0.002% of the sequences were unidentified. Proteobacteria was the most abundant phylum recovered by all three techniques, representing from 40% of sequences in the case of vacuum samples to 45% in the case of swab samples. The next three most abundant phyla were Actinobacteria, Firmicutes, and Bacteroidetes representing ~ 26, ~ 12, and ~ 10% of reads, respectively (Fig. 1).

In the case of fungal communities, after quality filtering, a total of 628,920 high-quality fungal sequences were clustered into 3012 OTUs. Median sequences generated per sample were 27,779, 36,982, and 6962 for vacuum, swab, and cut samples, respectively. All samples were rarefied to 2085 reads, yielding a total of 1569 distinct OTUs. In total, four fungal phyla were identified (Ascomycota, Basidiomycota, Chytridiomycota, and Zygomycota; Fig. 1). The class Dothideomycetes (phylum Ascomycota) was the most abundant recovered by all three techniques, representing from 60% of sequences in the case of vacuum samples. The next three most abundant classes were Agaricomycetes (Basidiomycota), Eurotiomycetes (Ascomycota), and Tremellomycetes (Basidiomycota). More than 80% of the sequences were captured by these four classes (Additional file 2: Figure S2).

**Fig. 2:**
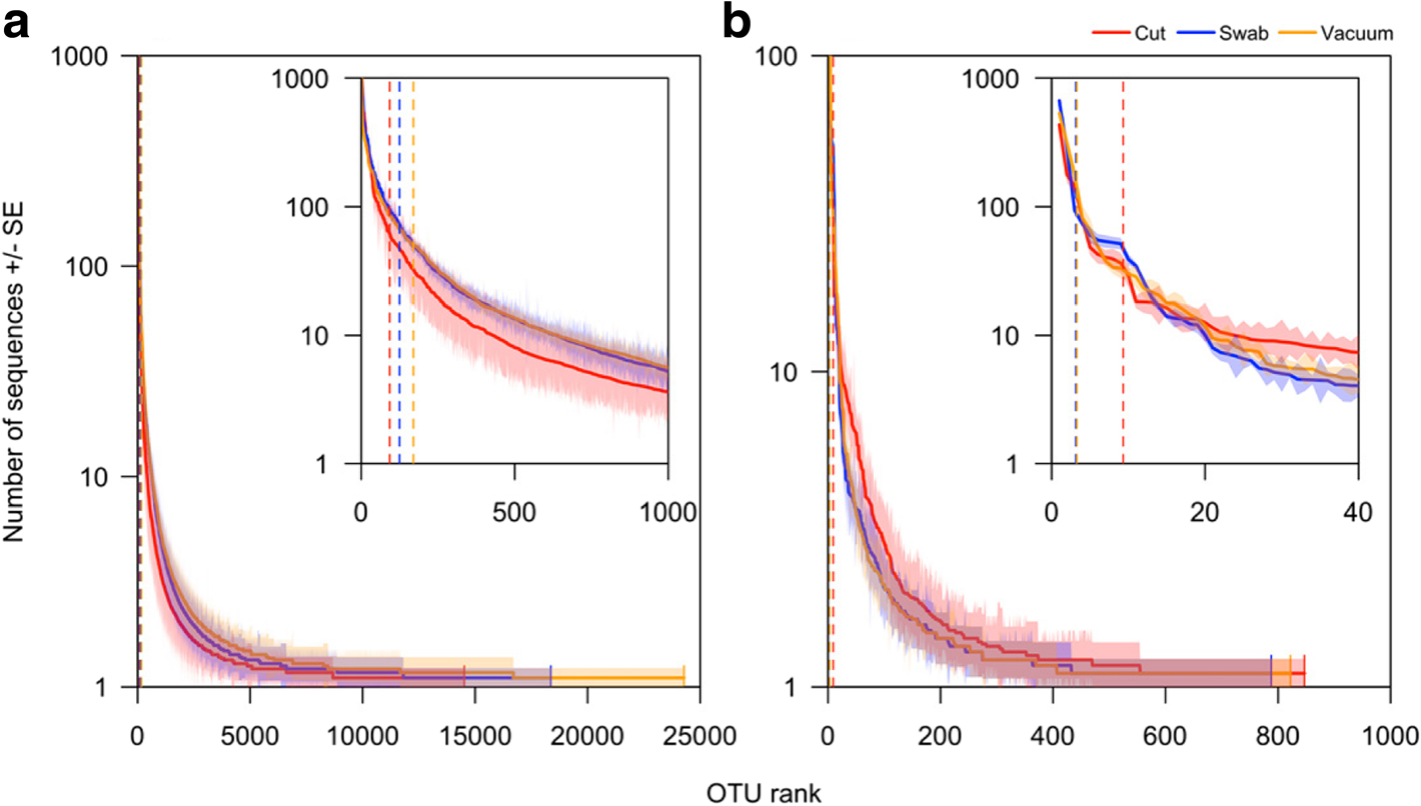
Taxon rank-abundance distributions for (**a**) bacteria and (**b**) fungi. Subplots magnify the initial portion of each curve. Rarefied OTU tables were log(*x* + 1)-transformed and mean abundances and standard errors within sample types (vacuums, swabs, and cuts) were computed for each OTU. OTUs were then ranked by mean abundance. Center lines represent mean abundances and shaded regions are standard errors based on seven samples per sample type. Dashed vertical lines represent the minimum number of OTUs that account for 50% of rarefied reads. For example, the red dashed vertical line in (**a**) shows that 93 bacterial OTUs accounted for 50% of all reads. For fungi, swab and vacuum 50% lines lie on top of each other. Note: *Y*-axes in both plots are log scale and were truncated at 1000. While not visible, abundance of bacterial OTUs ran as high as 3715 for bacteria and over 100 for fungi (geometric mean over seven samples)

To further elucidate alpha diversity patterns, taxon rank-abundance distributions were plotted for each of the three sample types (Fig. 2). OTU tables were log(x + 1)-transformed. OTUs were then ranked in order of mean abundance per sample type, and standard errors were computed for each OTU (*n* = 7 for each sample type). All sample types for both bacteria and fungi showed qualitatively similar long-tailed distributions. For bacteria, 50% of the rarefied reads were captured by 0.64, 0.68, and 0.70% of the OTUs for cut, swab, and vacuum samples, respectively. Fungal taxon distributions showed a slightly different trend, as cut samples produced a shorter tail than swab and vacuum samples. In this case, half of rarefied reads were accounted for by 1.1, 0.40, 0.40% of OTUs recovered by cut, swab, and vacuum samples, respectively.

With regard to OTU richness, bacterial and fungal communities again showed slightly differing trends. For bacteria, vacuum samples captured the greatest richness, followed by swab and cut samples (24,279, 18,363, and 14,518 OTUs, respectively, in the rarefied dataset). For fungi, however, sample type impacted richness less, as the number of OTUs recovered was similar for vacuum, swab, and cut samples (822, 788, and 847 OTUs, respectively). In all cases, the relatively high degree of richness reflects the long tail of these taxon rank-abundance distributions.

### Bacterial source attribution as a function of sample type

**Fig. 3:**
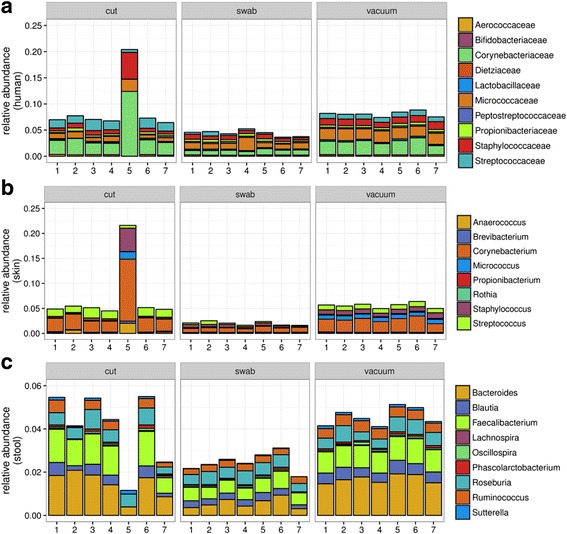
Bacterial source environment attribution for (**a**) human indicator bacterial families used in Meadows et al. [71], (**b**) skin indicator bacterial genera used in Barberan et al. [87], and (**c**) human stool indicator genera used in Barberan et al. [87]. The *y*-axis represents relative abundance of reads for a given sample

Bacterial community composition was further broken down into taxonomic groupings indicative of potential source environments. The 11 families previously identified as human indicators (Corynebacteriaceae, Staphylococcaceae, Streptococcaceae, Lactobacillaceae, Propionibacteriaceae, Peptostreptococcaceae, Bifidobacteriaceae, Micrococcaceae, Dietziaceae, Aerococcaceae, Tissierellaceae) [71] together accounted for 8.1% (standard error, SE = 0.2%), 4.4% (SE = 0.2%), and 8.9% (SE = 1.9%) of the reads for vacuum, swab, and cut samples, respectively (Fig. 3a). Skin indicator genera (*Propionibacterium*, *Staphylococcus*, *Corynebacterium*, *Streptococcus*, *Rothia*, *Micrococcus*, *Anaerococcus*, and *Brevibacterium*) [21] contributed a greater proportion of reads than any other indicator group assessed (Fig. 3b). While vacuum and cut samples showed similar contributions from skin-associated genera, 5.6% (SE = 0.2%) and 7.4% (SE = 2.4%), respectively, the same genera accounted for only 2.0% (SE = 0.1%) reads in swab samples. Stool-associated genera (*Bacteroides*, *Faecalibacterium*, *Lachnospira*, *Oscillospira*, *Roseburia*, *Coprococcus*, *Ruminococcus*, *Parabacteroides*, *Phascolarctobacterium*, *Sutterella*, and *Blautia*) [21] in vacuum and cut samples contributed an average of 4.6% (SE = 1.5%) and 4.1% (SE = 0.6%) of the reads, while 2.5% (SE = 0.2%) in swab samples (Fig. 3c). Bacteria potentially sourced from soil (Solibacteraceae, Chloracidobacteria, *Bradyrhizobium*, *Rhizobium*, among others), marine environments (Pelagibacteraceae, *Synechococcus*, *Marinobacter*, *Polaribacter*, among others), and insects (*Wolbachia*, *Buchnera*, *Rickettsiella*, *Blattabacterium*, *Baumannia*, among others) [21] were also detectable, although all were present in proportions more than an order of magnitude lower than the skin- and stool-associated genera.

### Fungal source attribution as a function of sample type

**Fig. 4:**
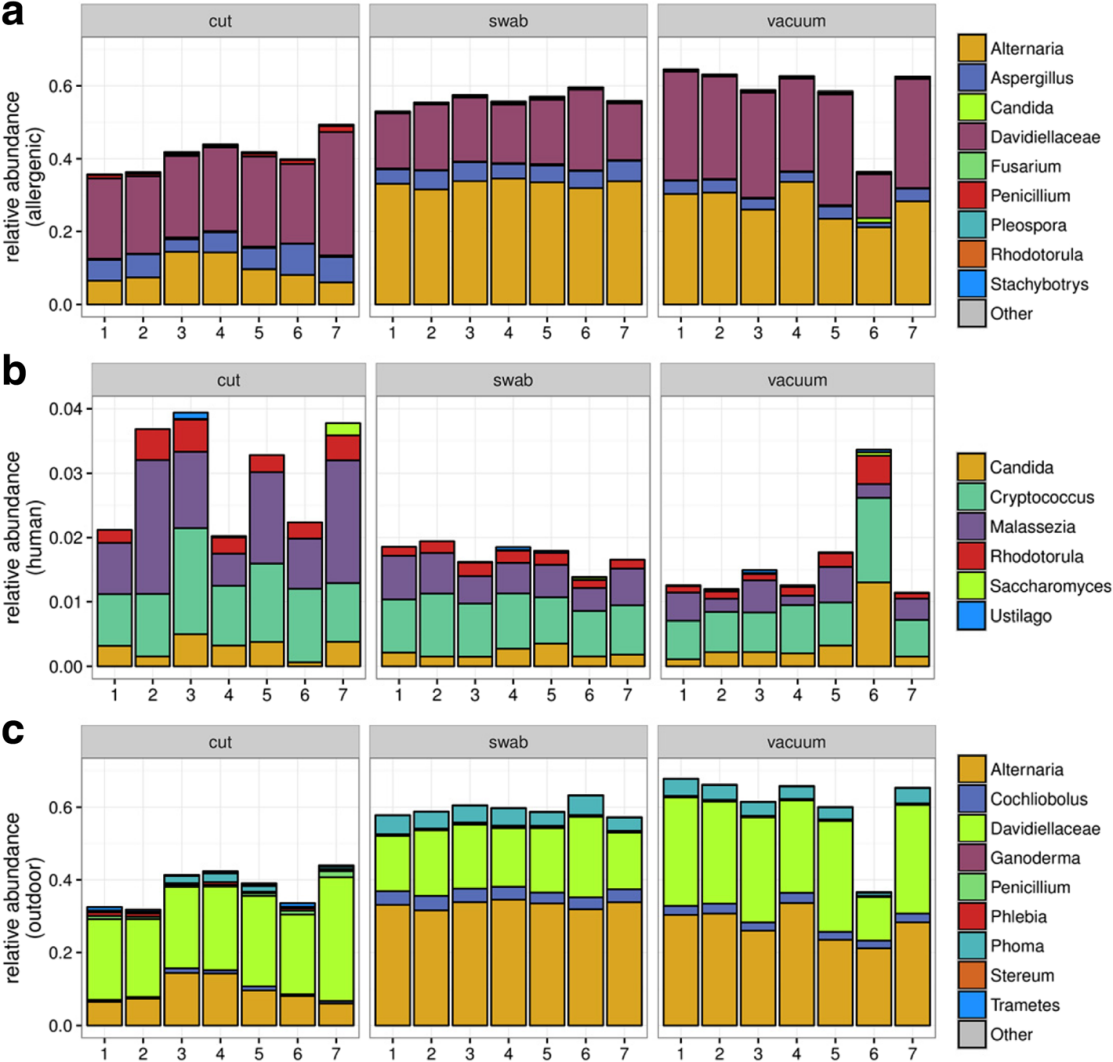
Fungal source environment attribution for (**a**) genera containing allergenic fungi [36, 88], (**b**) common fungal taxa from human skin [72], and (**c**) outdoor associated fungal taxa, including plant pathogens, wood rot and soil-associated taxa [21, 89]

With regard to fungal community composition, relevant genera containing allergenic species were recovered by all three sampling techniques (Fig. 4a). The genera *Alternaria* and *Aspergillus* and the family Davidiellaceae (which includes the genera *Cladosporium* and *Davidiella*) were the most prevalent across all three techniques. *Alternaria* was more predominant in swabs and vacuum samples than in cuts. Five genera previously found [72] to be human skin-associated (*Candida*, *Cryptococcus*, *Malassezia*, *Rhodotorula*, and *Saccharomyces*) were also found in the HVAC filter in relative abundances of 2 to 4% across all three techniques (Fig. 4b). Common outdoor-associated fungal taxa (wood rot, plant and soil associated) were recovered in high proportions by all three techniques.

### Community between-sample diversity

**Fig. 5:**
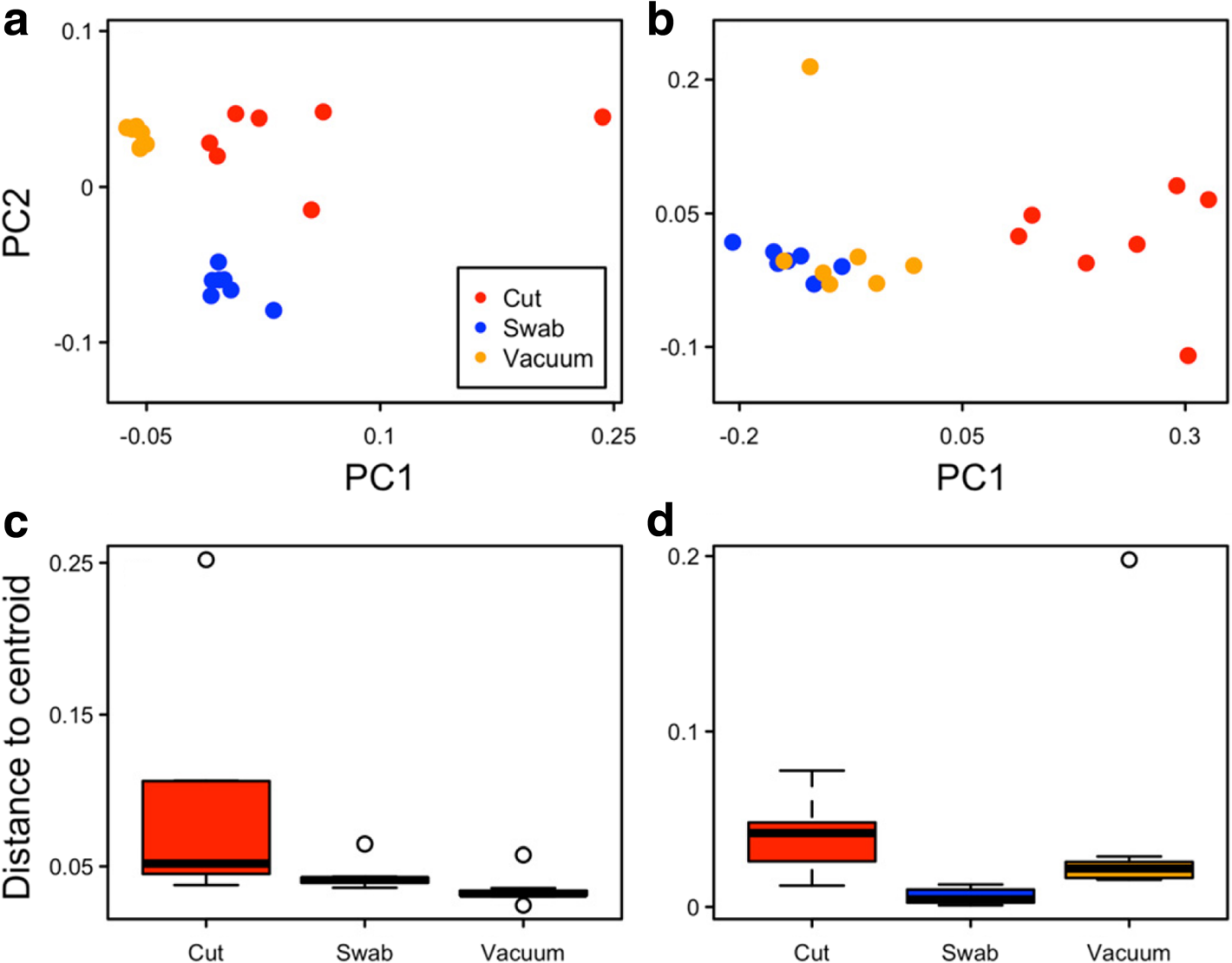
Community repeatability by sample type. PCoA plots for (**a**) bacteria (variance explained PC1 = 44% and PC2 = 22%) and (**b**) fungi (variance explained PC1 = 59% and PC2 = 9%) are based on weighted UniFrac and Morisita-Horn distances, respectively. Boxplots for (**c**) bacteria and (**d**) fungi represent distances to group centroids produced by the betadisper function in Vegan, which runs an analysis of multivariate homogeneity of dispersions

Distances between microbial assemblages collected from a single HVAC filter were visualized with boxplots of multivariate group dispersion by sample type and principal component analysis (PCoA) plots (Fig. 5). Communities were a priori grouped by sample type and analyzed with permutational analysis of dispersion and permutational analysis of variance (betadisper and ADONIS, respectively) based on weighted UniFrac distances [67] for bacteria and Morisita-Horn distances for fungi. As evident in Fig. 5a, the recovered bacterial assemblages clustered by sample method and assemblages recovered by swabbing and vacuuming were less variable than those recovered in cut samples (global *p* = 0.02, betadisper), although pairwise tests were not statistically significant (Tukey HSD, *p* > 0.05 for all comparisons). Sample type explained about half of the variation in distances between bacterial communities (ADONIS *R*^2^ = 0.48, *p* = 0.001 on 999 permutations).

For fungi, trends in community clustering were somewhat different (Fig. 5b). Swab and vacuum samples appear more similar to each other than to cuts, while group dispersions were not significantly different across sample types (betadisper, *p* = 0.11). In this case, sample type explained substantially more of the variation in distances between communities (ADONIS *R*^2^ = 0.92, *p* = 0.001). It should be noted that the relative statistical importance of sample type for fungi compared to bacteria is likely due in part to the fact that homoscedasticity of dispersions across sample types was met for fungi, but not for bacteria, as cited above.

**Fig. 6:**
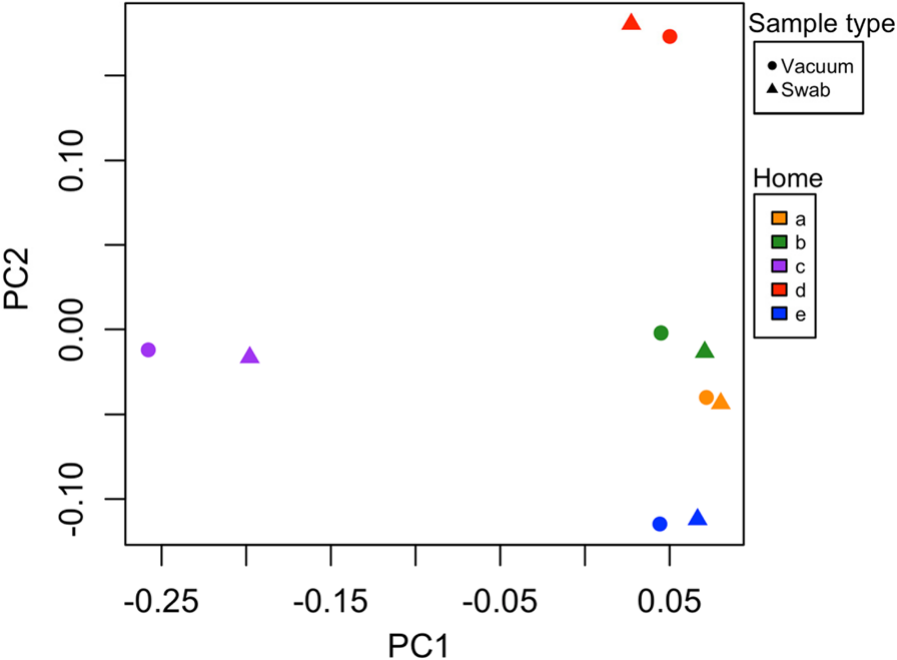
Bacterial community clustering by household and sample type (PCoA). Color denotes household. The orange points (house “a” in the legend) are from the house filter analyzed throughout this paper. Shape denotes sample type (circle = vacuum, triangle = swab). Cut samples and fungal sequences were not available for the multi-home analysis

To further understand the potential impact of sampling technique variation on bacterial communities recovered via the different sampling methods, swab and vacuum samples collected from HVAC filters in five different homes were compared. As evident in Fig. 6, bacterial communities clustered by household, rather than by sample type (either vacuum or swab). A permutational analysis of variance (ADONIS) confirmed this observation as household *R*^2^ = 0.77 (*p* = 0.001), while sample type was not a significant explanatory factor (*R*^2^ = 0.06, *p* > 0.05). It is expected that the results would be similar with the inclusion of cut samples, although they were not available for this analysis.

The core microbiome (core microbiota) concept was used to further assess the repeatability of each of the three techniques. Defined here as the OTUs shared by all samples of a given sample type (e.g., the OTUs that appear in all seven vacuum samples), the core microbiome describes shared OTU membership. For bacteria, the core microbiome of vacuum samples represented 6.2% of the total OTUs recovered in all vacuum samples. These OTUs accounted for 80% of the vacuum sample sequences obtained in the rarefied dataset. Similarly, the core microbiome for swab samples represented 6.3% of the total swab OTUs and 75% of the reads. Cut samples, however, recovered a smaller core comprising 1.6% of the total cut OTUs and 44% of the reads. Thus, vacuums and swabs shared higher percentages of OTUs and sequences between all their respective samples than did cuts. It is also interesting to note that out of 36,265 bacterial OTUs in the whole data set, only 6203 OTUs were common to all three sample types and 47% of all OTUs appeared only once.

For fungi, all three sample techniques performed similarly in terms of core community reproducibility. Samples shared 6.9, 7.4, and 8.2% of their OTUs, representing 79, 78, and 81% of the total reads for vacuum, swab, and cut samples, respectively. Similar to the bacterial data, 51% of all fungal OTUs appeared only once. Together, these numbers suggest that in all of these cases, for both bacteria and fungi, community structure is much more repeatable than community membership, which is in part attributable to the long-tail OTU rank-abundance distributions mentioned above. They also suggest that for fungi, all three sampling methodologies were similarly repeatable, while for bacteria, cut samples were less repeatable than swab or vacuum samples in terms of both community structure and membership.

To understand how well the communities captured by each sample type reflected the global HVAC community approximated by the entire dataset, simplified communities comprising only core OTUs were constructed for each of the three sample types. Weighted UniFrac distance matrices based on these three core communities were then correlated with the global distance matrix based on all OTUs in the whole dataset using a permutational Mantel test. Results indicated that all three core bacterial matrices were highly correlated with the overall distance matrix, although vacuum- and swab-derived cores reflected patterns in the overall dataset (Mantel *r* = 0.997 and 0.996, respectively; *p* = 0.001 based on 999 permutations) even more strongly than did cut-derived core bacteria (Mantel *r* = 0.95, *p* = 0.001). For fungi, the correlation between core OTU communities from each sample type and the global community was strong and of similar degree for vacuum, swab, and cut sample types (Mantel *r* = 0.97, 0.97, 0.98, respectively; *p* = 0.001) Thus, core OTUs drove patterns in community structure, while sporadically present OTUs contributed little to community structure for both bacteria and fungi.

## Discussion

### Quantitative DNA recovery

An important aspect when comparing environmental matrix processing techniques is their relative ability to recover biological material. In the case of HVAC filter dust, vacuum samples recovered more total, bacterial, and fungal DNA. Communities obtained from low biomass samples may be more prone to influence by background DNA noise coming from reagents or contamination introduced during sample processing [73]. Thus, even though DNA concentrations are normalized before sequencing, being able to recover higher amounts of bacterial and fungal DNA could represent an advantage.

In general, the genome copy numbers per gram of HVAC filter dust in vacuum samples are on the lower end of the range observed in previous work reporting DNA concentrations in floor dust [74]. Along with the difference in matrix, this may be due to the fact that in the present study, the dust was not sieved in order to obtain sufficient material from the HVAC filters for subsequent analysis. Thus, a larger portion of the measured mass was not biological material, but filter fibers and other inorganic dust fibers, which would decrease the mass concentration of DNA. In separate tests undertaken to estimate the dust to fiber recovery ratios for HVAC vacuum samples from the same filter type used in this work, it was observed that fibers contributed as much as 90% of the recovered mass (unpublished data). Accounting for this approximate 10× dilution factor yields a DNA density in this HVAC filter dust that is comparable to that obtained in sieved floor dust.

It is worth highlighting that sample technique significantly impacts the bacteria to fungi recovery ratio (ANOVA, *p* < 0.001). This ratio was higher in cuts than in vacuum and swab samples. This may be related to the combination of the hydrophobicity of fungal cell walls and the protocol for processing cut samples, which includes sonicating and vortexing in PBS solution. Conversely, vacuum samples do not use any pre-extraction solution and swab samples use only a small amount of PBS-tween solution to moisten the swab. Also, the prefiltration step in the cut sample method may have further affected the bacteria to fungi ratio since fungal particles such as conidial chains can be larger than the 20 μm and thus removed in the pre-filter.

### Within-sample diversity and variability

All sampling techniques revealed many taxa typical of the home microbiota. The dominant bacterial phyla captured by the analyzed HVAC filter are characteristic of home microbiota described previously [21, 29, 75]. The fraction of taxa associated with humans found in this HVAC dust supports the claim that humans are important sources of indoor airborne bacteria [71, 76–78]. Fungal communities recovered were found to be dominated by outdoor-associated taxa, whereas typical human skin-associated fungal taxa were found in low relative abundances. These findings are in agreement with previous studies that found outdoor taxa to be primary sources of indoor fungal communities [20, 21, 79]. All techniques recovered fungal allergenic taxa in similar proportions, except for the allergenic taxa *Alternaria*, which was recovered in lower relative abundance in cut samples. This could be related to the hydrophobicity of *Alternaria* external surfaces [80] and the use of PBS solution for sonication and vortexing during the processing of cut samples. Thus, it is possible that this technique could affect the fungal communities recovered from HVAC filters, favoring those fungal taxa with hydrophilic surfaces.

All three sampling techniques also produced qualitatively similar long-tailed OTU distributions. Previous studies have found that more abundant OTUs tend to appear more frequently across samples than less abundant OTUs, which is characteristic of a random sampling process [39, 51, 81–83]. These long-tailed OTU distributions help explain the high bacterial richness observed here, and the fact that a relatively low portion of OTUs and high portion of reads were shared within sample types. Thus, investigating the occurrence of rare taxa across space may be difficult, especially if sequencing depth is limited [84]. Novel statistical methods may be needed to understand the potential importance of rare taxa for bacterial community dynamics [85]. Given the higher repeatability of weighted measures of community structure, our findings suggest that environmental differences in community structure may be compared with greater confidence than differences in community membership.

### Between-sample diversity and variability

Our results indicate that vacuum and swab samples of HVAC filter dust were more repeatable than cut samples in terms of both community structure and membership for both bacteria and fungi. Community structure was more consistent across vacuum and swab samples as indicated by smaller weighted UniFrac and Morisita-Horn distances, respectively, and larger abundance-based core communities. Larger membership-based core OTU communities for these two sample types compared to cut samples indicate that their community membership was also more consistent. Also, given that the bacteria vacuum and swab core communities were more closely correlated to the global communities sampled, vacuum, and swab HVAC filter samples appear to produce more representative bacterial HVAC samples.

Our study was primarily designed to assess the repeatability of three representative HVAC dust sampling methods by examining replicate environmental samples. Since sample type proved to be a significant explanatory factor of community differences, we sought to understand the relative importance of these sample type-produced community differences in a broader context. Employing swab and vacuum samples from HVAC filters in five homes, we found that bacterial communities strongly clustered by household, with relatively minor variability attributable to sampling method. This result suggests that while mixing these sample methodologies in a larger scale study would likely introduce noise, it would still be possible to distinguish the airborne microbiota between households by sampling the HVAC filters in climate-controlled homes. However, since our study only captured a snapshot of the microbial communities present in these five homes, further research would be required to assess how the time of year might affect household-level clustering.

### Sample processing considerations

Practical advantages of HVAC filter sampling for indoor airborne microbiome analysis include the relative ease of installation and lack of intrusiveness during long-term sampling periods. Developing sampling protocols to remove dust from the filters depends not only on microbiological considerations, but also on the relative cost, and labor required for each method. When considering various passive samplers for airborne microbiota characterization in homes, one study found that microbial communities were little affected by sampler type when compared to effects from different environments, and thus, ease of sample collection and economics were likely to be the primary drivers of sampling protocol selection [39]. In our study, cut sampling was more labor intensive than vacuum and swab sampling, since cutting HVAC filters is physically difficult, and the following elution and filtration steps require additional time. Thus, cut sampling is less desirable based on both bacterial community repeatability and practical considerations.

### Limitations

A limitation of this study is that the repeatability of these sampling methods was primarily assessed by an in-depth analysis of a single HVAC filter. It is possible that repeatability could vary across different filters, across seasons, or in different homes. Another limitation is that the mass recovered from the filter for cut and swab methods was not determined due to the difficulty in accurately assessing the mass recovered via the liquid extraction method employed.

As is always the case when studying complex media such as HVAC dust, it was impossible to completely eliminate environmental variability across replicate samples. Nevertheless, this study was designed to minimize environmental variability by compositing dust from five randomly chosen locations on a single filter for each sample. Previously, it was shown for home floor dust that even when this spatial homogenization was not performed (i.e., adjacent 1-m^2^ areas were vacuumed) bacterial communities were still highly concordant [86]. Thus, it is assumed that environmental variability was minimal in the present study.

## Conclusions

Sampling methodology can affect the recovery, repeatability, structure, and membership of microbial communities recovered from dust samples in the built environment. All three HVAC dust sampling techniques evaluated in the current study yielded microbial communities consistent with previous studies of occupied homes with bacterial communities reflecting human occupancy and fungal communities reflecting outdoor-associated taxa. However, the results suggest that vacuum and swab samples of HVAC filter dust recover greater quantities of DNA and produce more repeatable microbial communities than cut samples. The vacuum and swab samples also yielded bacterial communities with greater richness than cut samples although all three techniques yielded fungal communities of similar richness. For all sample types, a small number of OTUs represented a significant fraction of the sequences. However, nearly 50% of all fungal and bacterial OTUs appeared only once in the sample set. Thus, in these dust samples, community membership (e.g., of rare taxa) is much less consistent than the core microbiome structure of the microbial communities recovered. Interestingly, bacterial communities recovered from repeated sampling of a single HVAC filter clustered by sampling technique with swab and vacuum samples yielding the most consistent results. However, a comparison of swab and vacuum samples from different homes in the same geographical area indicates that bacterial communities cluster more strongly by household than by sample type. While the results of this study are directly applicable to indoor microbiota studies utilizing HVAC filter dust, they are also valuable for evaluating other common sampling techniques (e.g., swabbing and vacuuming) used to collect dust from other indoor surfaces such as floors. More broadly, the results help define the variability inherent in the microbiota inferred from dust samples collected from indoor environments.

## Additional files

Additional file 1: Figure S1. Bacteria to fungi genome copy numbers ratio for the three techniques evaluated *n* = 7 per technique. (DOCX 65 kb)

Additional file 2: Figure S2. Relative abundance of top six fungal classes per sample. (DOCX 294 kb)

## References

[1] Adgate JL, Church TR, Ryan AD, Ramachandran G, Fredrickson AL, Stock TH, Morandi MT, Sexton K (2004). Outdoor, indoor, and personal exposure to VOCs in children. Environ Health Perspect.

[2] Franck U, Herbarth O, Wehner B, Wiedensohler A, Manjarrez M (2003). How do the indoor size distributions of airborne submicron and ultrafine particles in the absence of significant indoor sources depend on outdoor distributions?. Indoor Air.

[3] Klepeis NE, Nelson WC, Ott WR, Robinson JP, Tsang AM, Switzer P, Behar JV, Hern SC, Engelmann WH (2001). The National Human Activity Pattern Survey (NHAPS): a resource for assessing exposure to environmental pollutants. J Expo Anal Environ Epidemiol.

[4] Andelman JB (1985). Organic micropollutants in drinking water and health inhalation exposure in the home to volatile organic contaminants of drinking water. Sci Total Environ.

[5] Berglund B, Brunekreef B, Knöppe H, Lindvall T, Maroni M, Mølhave L, Skov P (1992). Effects of indoor air pollution on human health. Indoor Air.

[6] Bernstein JA, Alexis N, Bacchus H, Bernstein IL, Fritz P, Horner E (2008). The health effects of nonindustrial indoor air pollution. J Allergy Clin Immunol.

[7] Fung F, Hughson WG (2003). Health effects of indoor fungal bioaerosol exposure. Appl Occup Environ Hyg.

[8] Hardin BD, Kelman BJ, Saxon A (2003). Adverse human health effects associated with molds in the indoor environment. J Occup Env Med.

[9] Jones AP (1999). Indoor air quality and health. Atmos Environ.

[10] Lax S, Nagler CR, Gilbert JA (2015). Our interface with the built environment: immunity and the indoor microbiota. Trends Immunol.

[11] Spengler JD, Sexton K (1983). Indoor air pollution: a public health perspective. Science.

[12] Stolwijk JAJ (1992). Risk assessment of acute health and comfort effects of indoor air pollution. Ann N Y Acad Sci.

[13] Wu N, Herrmann T, Paepke O, Tickner J, Hale R, Harvey E, La Guardia M, McClean MD, Webster TF (2007). Human exposure to PBDEs: associations of PBDE body burdens with food consumption and house dust concentrations. Environ Sci Technol.

[14] Bartram AK, Lynch MDJ, Stearns JC, Moreno-Hagelsieb G, Neufeld JD (2011). Generation of multimillion-sequence 16S rRNA gene libraries from complex microbial communities by assembling paired-end Illumina reads. Appl Environ Microbiol.

[15] Margulies M, Egholm M, Altman WE, Attiya S, Bader JS, Bemben LA (2005). Genome sequencing in microfabricated high-density picolitre reactors. Nature.

[16] Quail MA, Smith M, Coupland P, Otto TD, Harris SR, Connor TR, Bertoni A, Swerdlow HP, Gu Y (2012). A tale of three next generation sequencing platforms: comparison of Ion Torrent, Pacific Biosciences and Illumina MiSeq sequencers. BMC Genomics.

[17] Shendure J, Ji H (2008). Next-generation DNA sequencing. Nat Biotechnol.

[18] Shendure J, Porreca GJ, Reppas NB, Lin X, McCutcheon JP, Rosenbaum AM, Wang MD, Zhang K, Mitra RD, Church GM (2005). Accurate multiplex polony sequencing of an evolved bacterial genome. Science.

[19] Adams RI, Miletto M, Lindow SE, Taylor JW, Bruns TD (2014). Airborne bacterial communities in residences: similarities and differences with fungi. PLoS One.

[20] Adams RI, Miletto M, Taylor JW, Bruns TD (2013). Dispersal in microbes: fungi in indoor air are dominated by outdoor air and show dispersal limitation at short distances. ISME J.

[21] Barberan A, Dunn RR, Reich BJ, Pacifici K, Laber EB, Menninger HL, Morton JM, Henley JB, Leff JW, Miller SL, Fierer N. The ecology of microscopic life in household dust. Proc Biol Sci. 2015;282(1814):​20151139. 10.1098/rspb.2015.1139 PMC4571696 26311665

[22] Corsi RL, Kinney KA, Levin H (2012). Microbiomes of built environments: 2011 symposium highlights and workgroup recommendations. Indoor Air.

[23] Dunn RR, Fierer N, Henley JB, Leff JW, Menninger HL (2013). Home life: factors structuring the bacterial diversity found within and between homes. PLoS One.

[24] Fierer N, Lauber CL, Zhou N, McDonald D, Costello EK, Knight R (2010). Forensic identification using skin bacterial communities. PNAS.

[25] Flores GE, Bates ST, Caporaso JG, Lauber CL, Leff JW, Knight R, Fierer N (2013). Diversity, distribution and sources of bacteria in residential kitchens. Environ Microbiol.

[26] Kelley ST, Gilbert JA (2013). Studying the microbiology of the indoor environment. Genome Biol.

[27] Kembel SW, Jones E, Kline J, Northcutt D, Stenson J, Womack AM, Bohannan BJM, Brown GZ, Green JL (2012). Architectural design influences the diversity and structure of the built environment microbiome. ISME J.

[28] Kembel SW, Meadow JF, O’Connor TK, Mhuireach G, Northcutt D, Kline J, Moriyama M, Brown GZ, Bohannan BJM, Green JL (2014). Architectural design drives the biogeography of indoor bacterial communities. PLoS One.

[29] Lax S, Smith DP, Hampton-Marcell J, Owens SM, Handley KM, Scott NM (2014). Longitudinal analysis of microbial interaction between humans and the indoor environment. Science.

[30] Luongo JC, Barberan A, Hacker-Cary R, Morgan EE, Miller SL, Fierer N (2017). Microbial analyses of airborne dust collected from dormitory rooms predict the sex of occupants. Indoor Air.

[31] Meadow JF, Altrichter AE, Kembel SW, Kline J, Mhuireach G, Moriyama M (2014). Indoor airborne bacterial communities are influenced by ventilation, occupancy, and outdoor air source. Indoor Air.

[32] Meadow JF, Altrichter AE, Kembel SW, Moriyama M, O’Connor TK, Womack AM, Brown GZ, Green JL, Bohannan BJM (2014). Bacterial communities on classroom surfaces vary with human contact. Microbiome.

[33] Peccia J, Hospodsky D, Bibby K (2011). New directions: a revolution in DNA sequencing now allows for the meaningful integration of biology with aerosol science. Atmos Environ.

[34] Dannemiller KC, Mendell MJ, Macher JM, Kumagai K, Bradman A, Holland N, Harley K, Eskenazi B, Peccia J (2014). Next-generation DNA sequencing reveals that low fungal diversity in house dust is associated with childhood asthma development. Indoor Air.

[35] Ege MJ, Mayer M, Normand A-C, Genuneit J, Cookson WOCM, Braun-Fahrländer C, Heederik D, Piarroux R, von Mutius E (2011). Exposure to environmental microorganisms and childhood asthma. N Engl J Med.

[36] Dannemiller KC, Gent JF, Leaderer BP, Peccia J (2016). Indoor microbial communities: influence on asthma severity in atopic and nonatopic children. J Allergy Clin Immunol.

[37] Fujimura KE, Demoor T, Rauch M, Faruqi AA, Jang S, Johnson CC, Boushey HA, Zoratti E, Ownby D, Lukacs NW, Lynch SV (2014). House dust exposure mediates gut microbiome *Lactobacillus* enrichment and airway immune defense against allergens and virus infection. PNAS.

[38] Noris F, Siegel JA, Kinney KA (2011). Evaluation of HVAC filters as a sampling mechanism for indoor microbial communities. Atmos Environ.

[39] Adams RI, Tian Y, Taylor JW, Bruns TD, Hyvärinen A, Täubel M (2015). Passive dust collectors for assessing airborne microbial material. Microbiome.

[40] Haaland D, Siegel JA (2017). Quantitative filter forensics for indoor particle sampling. Indoor Air.

[41] Gurevich I, Tafuro P, Krystofiak SP, Kalter RD, Cunha BA (1984). Three clusters of Bacillus pseudobacteremia related to a radiometric blood culture analyzer. Infect Control.

[42] Elixmann JH, Jorde W, Linskens HF (1987). Filters of an air-conditioning installation as disseminators of fungal spores. Experientia Suppl.

[43] Noris F, Siegel JA, Kinney KA (2009). Biological and metal contaminants in HVAC filter dust. Ashrae Tran.

[44] Batterman S, Godwin C, Chernyak S, Jia C, Charles S (2010). Brominated flame retardants in offices in Michigan, USA. Environ Int.

[45] Stanley NJ, Kuehn TH, Kim SW, Raynor PC, Anantharaman S, Ramakrishnan MA, Goyal SM (2008). Background culturable bacteria aerosol in two large public buildings using HVAC filters as long term, passive, high-volume air samplers. J Environ Monit.

[46] Moore CE, McCarthy R, Logsdon RF. A partial chemical analysis of atmospheric dirt collected for study of soiling properties. Heat Piping Air Cond. 1954;(October):145–8.

[47] Hoisington A, Maestre JP, King MD, Siegel JA, Kinney KA (2014). The impact of sampler selection on characterizing the indoor microbiome. Build Environ.

[48] Emerson JB, Keady PB, Brewer TE, Clements N, Morgan EE, Awerbuch J, Miller SL, Fierer N (2015). Impacts of flood damage on airborne bacteria and fungi in homes after the 2013 Colorado Front Range Flood. Environ Sci Technol.

[49] Barnes CS, Allenbrand R, Mohammed M, Gard L, Pacheco F, Kennedy K, Portnoy JM, Ciaccio C (2015). Measurement of aeroallergens from furnace filters. Ann Allergy Asthma Immunol.

[50] Hoisington A, Maestre JP, Siegel JA, Kinney KA (2014). Exploring the microbiome of the built environment: a primer on four biological methods available to building professionals. HVAC&R Res.

[51] Fahlgren C, Bratbak G, Sandaa R-A, Thyrhaug R, Zweifel UL (2010). Diversity of airborne bacteria in samples collected using different devices for aerosol collection. Aerobiologia.

[52] Frankel M, Timm M, Hansen EW, Madsen AM (2012). Comparison of sampling methods for the assessment of indoor microbial exposure. Indoor Air.

[53] Checinska A, Probst AJ, Vaishampayan P, White JR, Kumar D, Stepanov VG, Fox GE, Nilsson HR, Pierson DL, Perry J, Venkateswaran K (2015). Microbiomes of the dust particles collected from the International Space Station and spacecraft assembly facilities. Microbiome.

[54] Lauber CL, Zhou N, Gordon JI, Knight R, Fierer N (2010). Effect of storage conditions on the assessment of bacterial community structure in soil and human-associated samples. FEMS Microbiol Lett.

[55] Li C-S, Lin Y-C (2001). Storage effects on bacterial concentration: determination of impinger and filter samples. Sci Total Environ.

[56] Baker GC, Smith JJ, Cowan DA (2003). Review and re-analysis of domain-specific 16S primers. J Microbiol Methods.

[57] Wang Y, Qian P-Y (2009). Conservative fragments in bacterial 16S rRNA genes and primer design for 16S ribosomal DNA amplicons in metagenomic studies. PLoS One.

[58] Gardes M, Bruns TD (1993). ITS primers with enhanced specificity for basidiomycetes—application to the identification of mycorrhizae and rusts. Mol Ecol.

[59] White TJ, Bruns T, Lee S, Taylor J. Amplification and direct sequencing of fungal ribosomal RNA genes for phylogenetics. In: Innis MA, Gelfand DH, Sninsky JJ, White TJ, editors. PCR protocols: a guide to methods and applications. Academic Press; 1990. p. 315–22.

[60] Caporaso JG, Kuczynski J, Stombaugh J, Bittinger K, Bushman FD, Costello EK (2010). QIIME allows analysis of high-throughput community sequencing data. Nat Methods.

[61] Dannemiller KC, Reeves D, Bibby K, Yamamoto N, Peccia J (2014). Fungal High-throughput Taxonomic Identification tool for use with Next-Generation Sequencing (FHiTINGS). J Basic Microbiol.

[62] Magoč T, Salzberg SL (2011). FLASH: fast length adjustment of short reads to improve genome assemblies. Bioinformatics.

[63] Edgar RC (2010). Search and clustering orders of magnitude faster than BLAST. Bioinformatics.

[64] Wang Q, Garrity GM, Tiedje JM, Cole JR (2007). Naive Bayesian classifier for rapid assignment of rRNA sequences into the new bacterial taxonomy. Appl Environ Microbiol.

[65] McDonald D, Price MN, Goodrich J, Nawrocki EP, TZ DS, Probst A, Andersen GL, Knight R, Hugenholtz P (2012). An improved Greengenes taxonomy with explicit ranks for ecological and evolutionary analyses of bacteria and archaea. ISME J.

[66] Kõljalg U, Nilsson RH, Abarenkov K, Tedersoo L, Taylor AFS, Bahram M (2013). Towards a unified paradigm for sequence-based identification of fungi. Mol Ecol.

[67] Lozupone C, Knight R (2005). UniFrac: a new phylogenetic method for comparing microbial communities. Appl Environ Microbiol.

[68] Oksanen J (2016). Multivariate analysis of ecological communities in R: vegan tutorial. R package version 2.3–3.

[69] Harms G, Layton AC, Dionisi HM, Gregory IR, Garrett VM, Hawkins SA, Robinson KG, Sayler GS (2003). Real-time PCR quantification of nitrifying bacteria in a municipal wastewater treatment plant. Environ Sci Technol.

[70] Zhou G, Whong WZ, Ong T, Chen B (2000). Development of a fungus-specific PCR assay for detecting low-level fungi in an indoor environment. Mol Cell Probes.

[71] Meadow JF, Altrichter AE, Bateman AC, Stenson J, Brown GZ, Green JL, Bohannan BJM (2015). Humans differ in their personal microbial cloud. PeerJ.

[72] Findley K, Oh J, Yang J, Conlan S, Deming C, Meyer JA, Schoenfeld D, Nomicos E, Park M, Kong HH, Segre JA (2013). Topographic diversity of fungal and bacterial communities in human skin. Nature.

[73] Salter SJ, Cox MJ, Turek EM, Calus ST, Cookson WO, Moffatt MF, Turner P, Parkhill J, Loman NJ, Walker AW (2014). Reagent and laboratory contamination can critically impact sequence-based microbiome analyses. BMC Biol.

[74] Dannemiller KC, Gent JF, Leaderer BP, Peccia J (2015). Influence of housing characteristics on bacterial and fungal communities in homes of asthmatic children. Indoor Air.

[75] Miletto M, Lindow SE (2015). Relative and contextual contribution of different sources to the composition and abundance of indoor air bacteria in residences. Microbiome.

[76] Hospodsky D, Qian J, Nazaroff WW, Yamamoto N, Bibby K, Rismani-Yazdi H, Peccia J (2012). Human occupancy as a source of indoor airborne bacteria. PLoS One.

[77] Hospodsky D, Yamamoto N, Nazaroff WW, Miller D, Gorthala S, Peccia J (2015). Characterizing airborne fungal and bacterial concentrations and emission rates in six occupied children's classrooms. Indoor Air.

[78] Täubel M, Rintala H, Pitkäranta M, Paulin L, Laitinen S, Pekkanen J, Hyvärinen A, Nevalainen A (2009). The occupant as a source of house dust bacteria. J Allergy Clin Immunol.

[79] Shelton BG, Kirkland KH, Flanders WD, Morris GK (2002). Profiles of airborne fungi in buildings and outdoor environments in the United States. Appl Environ Microbiol.

[80] Chau HW, Si BC, Goh YK, Vujanovic V (2009). A novel method for identifying hydrophobicity on fungal surfaces. Mycol Res.

[81] Bowen JL, Morrison HG, Hobbie JE, Sogin ML (2012). Salt marsh sediment diversity: a test of the variability of the rare biosphere among environmental replicates. ISME J.

[82] Flores GE, Caporaso JG, Henley JB, Rideout JR, Domogala D, Chase J (2014). Temporal variability is a personalized feature of the human microbiome. Genome Biol.

[83] Yamamoto N, Dannemiller KC, Bibby K, Peccia J (2014). Identification accuracy and diversity reproducibility associated with internal transcribed spacer-based fungal taxonomic library preparation. Environ Microbiol.

[84] Knight R, Jansson J, Field D, Fierer N, Desai N, Fuhrman JA (2012). Unlocking the potential of metagenomics through replicated experimental design. Nat Biotechnol.

[85] Shade A, Jones SE, Caporaso JG, Handelsman J, Knight R, Fierer N, Gilbert JA (2014). Conditionally rare taxa disproportionately contribute to temporal changes in microbial diversity. MBio.

[86] Fujimura KE, Rauch M, Matsui E, Iwai S, Calatroni A, Lynn H (2012). Development of a standardized approach for environmental microbiota investigations related to asthma development in children. J Microbiol Methods.

[87] Barberan A, Ladau J, Leff JW, Pollard KS, Menninger HL, Dunn RR, Fierer N (2015). Continental-scale distributions of dust-associated bacteria and fungi. PNAS.

[88] Yamamoto N, Bibby K, Qian J, Hospodsky D, Rismani-Yazdi H, Nazaroff WW, Peccia J (2012). Particle-size distributions and seasonal diversity of allergenic and pathogenic fungi in outdoor air. ISME J.

[89] Adams RI, Miletto M, Taylor JW, Bruns TD. The diversity and distribution of fungi on residential surfaces. PLoS One. 2013;8(11):​e78866. 10.1371/journal.pone.0078866 PMC3815347 24223861

