# Supplementary figures and images for: Filter forensics: microbiota recovery from residential HVAC filters
Source: Microbiome. 2018 Jan 30;6:22. doi: 10.1186/s40168-018-0407-6 (PMC5791358; doi:10.1186/s40168-018-0407-6)

**
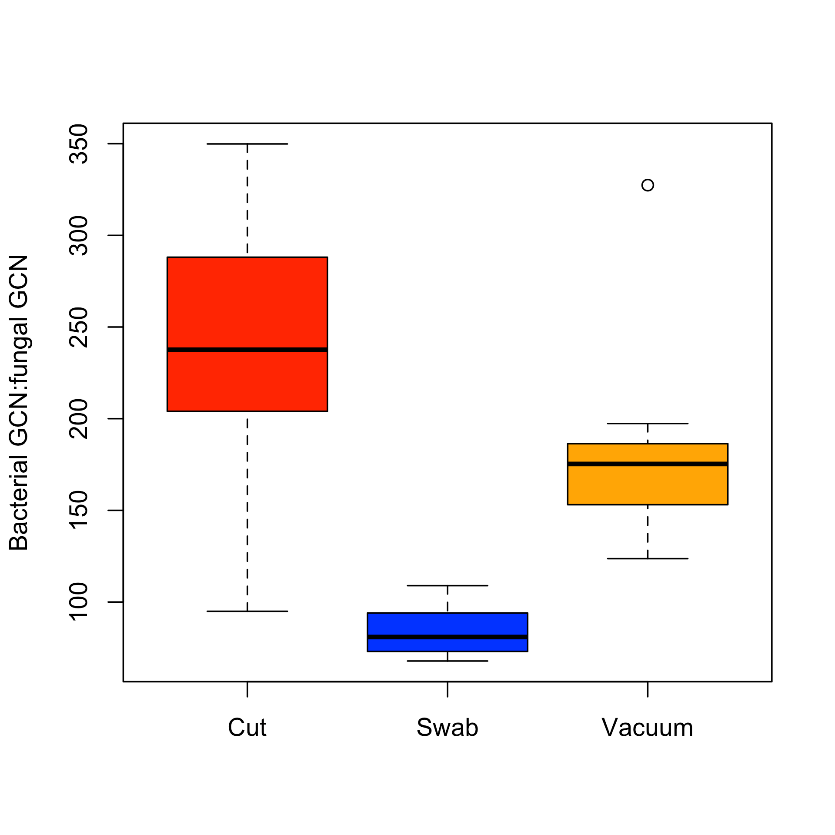
**

Supplement: Supplementary file 1 — Bacteria to fungi genome copy numbers ratio for the three techniques evaluated n = 7 per technique. (DOCX 65 kb) [file 40168_2018_407_MOESM1_ESM.docx]

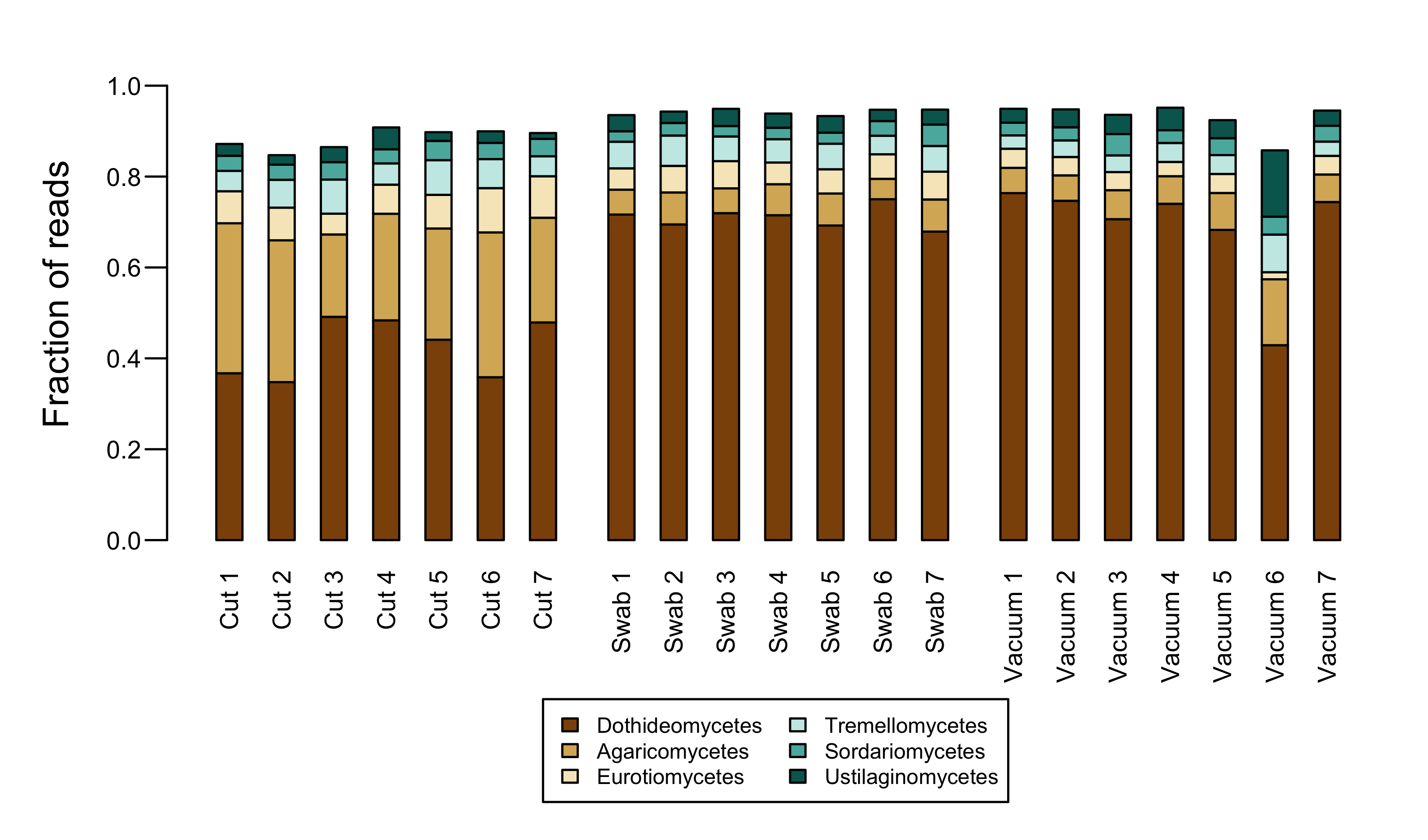

Supplement: Supplementary file 2 — Relative abundance of top six fungal classes per sample. (DOCX 294 kb) [file 40168_2018_407_MOESM2_ESM.docx]
